# Non-destructive measurement of egg yolk weight and percentage based on magnetic resonance imaging

**DOI:** 10.1016/j.psj.2025.105896

**Published:** 2025-09-23

**Authors:** Bingxin Luo, Lin Xuan, Honglei Jin, Yuanhang Shi, Jiahui Lai, Runzhe Wang, Longyu Zhuang, Feiyu Chen, Jiajie Yang, Wenbin Zhou, Anning Huang, Guiyun Xu, Jiangxia Zheng

**Affiliations:** National Engineering Laboratory for Animal Breeding and MOA Key Laboratory of Animal Genetics and Breeding, College of Animal Science and Technology, China Agricultural University, Beijing, 100193 China

**Keywords:** Yolk percentage, Non-destructive testing, Magnetic resonance imaging, Regression modeling, Hatching performance

## Abstract

With the expanding egg processing industry and increasing demand for egg yolk powder, efficient non-destructive methods for detecting yolk percentage have garnered significant attention. Existing non-destructive testing techniques frequently exhibit limited accuracy for brown eggs. To establish the optimal setup for non-destructive yolk measurement, we compared magnetic resonance imaging (MRI) field strengths and found that 3.0 T provided the best performance. Building on this, we established a standardized imaging workflow using 3D Slicer software, enabling non-destructive measurement of yolk volume and other relevant parameters. To build a robust predictive model, we then scanned 360 white eggs and 750 brown eggs, isolating the yolk via image segmentation algorithms to calculate parameters such as yolk volume, surface area, and Feret’s diameter. Using a 70/30 dataset split, the best-performing model achieved high coefficients of determination (r²) of 0.893 and 0.907 in the training and test sets, respectively, demonstrating excellent predictive accuracy. The model’s utility was further demonstrated by its ability to accurately predict yolk weight and percentage under varying conditions, including different shell colors and storage times. Analysis using the model revealed significantly lower yolk weight and percentage in Rhode Island Red (RIR) brown eggs compared to White Leghorn (WL) white eggs (*P* < 0.001), and long-term storage significantly increased these parameters (*P* < 0.001). Genetic analysis of RIR eggs also yielded heritability estimates of 0.39 for yolk weight and 0.42 for yolk percentage. Regarding safety, MRI exposure had no significant effect on hatchability, with a rate of 93.3 % in the treated group compared to 86.7 % in the control group (*P* > 0.05). This study provides an effective solution for rapid, non-destructive measurement of yolk percentage, which will significantly benefit layer production and ultimately support the development of the egg processing industry.

## INTRODUCTION

Eggs are a globally consumed nutrient-rich food that provides high-quality protein, essential vitamins, minerals, and play a vital role in the human diet (Réhault-Godbert et al., 2019). Egg yolk contains comprehensive nutrients, such as lipids, vitamins A, D, E and B group, and minerals such as calcium, iron and phosphorus. In recent years, the demand for egg products has shown structural growth (Yang et al., 2024; Shi et al., 2024). According to statistics, in 2023, China's total exports of processed eggs reached 29480.31 tons, a year-on-year increase of 5.66%, among which the export volume of yolk powder increased significantly, up 8.96% year-on-year (Liu et al., 2024b). This change has placed higher demands on layer breeding, making the development of varieties with high yolk weight and yolk percentage a key direction in the poultry industry.

The importance of the yolk extends beyond nutrition; it is also the sole energy source for embryonic development in fertilized eggs, providing 90% to 94% of the energy, essential lipids, and proteins for growth, as well as passive immunity through Immunoglobulin Y (IgY) (Schechtman and Knight, 1955). This dual role makes high yolk percentage a critical trait not only for the food industry but also for poultry breeding programs.

Yolk percentage, the proportion of yolk weight to whole egg weight, is an important indicator of egg quality. Significant differences in yolk percentage exist among eggs from different chicken breeds and laying stages, with heritability estimates ranging from 0.2 to 0.5, reflecting obvious genetic characteristics (Zhang et al., 2023, 2024). However, the traditional method of yolk percentage determination requires breaking eggs, which not only prevents the hatching of breeding eggs, but is also labor-intensive and inefficient. Therefore, it is important to develop efficient and non-destructive yolk percentage assay methods.

In order to solve the limitations of traditional method, dielectric, machine vision, spectroscopic and hyperspectral imaging techniques have been successively applied in the field of non-destructive testing of egg yolk percentage (Harnsoongnoen and Jaroensuk, 2021; Yuan et al., 2023). For example, dielectric-machine vision techniques reduced the yolk weight detection error to 4.24% for white-shelled eggs (Soltani et al., 2015); visible/near-infrared spectroscopy combined with image analysis achieved an R² of 0.82 for yolk weight prediction in duck eggs (Liu et al., 2024a). These techniques have disadvantages such as high environmental sensitivity, light source fluctuation, eggshell stains and other factors that can easily interfere with the detection accuracy of the machine vision and spectral methods (Ahmed et al., 2025). Moreover, the model generalization ability is insufficient, the dielectric method is sensitive to the moisture content, and the optical difference between the white-shelled eggs and brown-shelled eggs results in limited detection accuracy and universality (Qi et al., 2020).

The present study proposes a new method for yolk percentage prediction based on Magnetic Resonance Imaging (MRI). Unlike traditional techniques that rely on surface features or a single parameter, MRI enables three-dimensional (3D) volumetric reconstruction and large-scale sample modeling, offering new possibilities for accurate and non-destructive detection of yolk percentage. In recent years, MRI technology has gradually transitioned from precision laboratory use to industrial application. In the field of poultry egg testing, its feasibility and innovation have been validated by multiple studies. For example, MRI has been used to localize the embryonic disc in pre-hatching fertilized eggs (Klein et al., 2002), non-invasively assess eye development in chicken embryos (Lindner et al., 2017), and achieve non-invasive, real-time sex identification of 12-day-old embryos by the German imaging company Orbem (Mcdougal, 2023).

Magnetic resonance imaging (MRI) technology relies on the behavior of protons in a static magnetic field. When excited by radiofrequency pulses, these protons undergo energy level transitions and emit signals during relaxation processes to generate images. Unlike techniques requiring ionizing radiation or contrast agents, MRI provides inherent biological safety advantages (Ciampa et al., 2010; Pathmanaban et al., 2019). MRI imaging characteristics allow it to be free from the interference of eggshells, and thus it shows great potential in the field of non-destructive testing of the quality of eggs as well (Hutchison et al., 1992; Chen et al., 2023). In MRI of eggs, the marked difference in water content between yolk (∼50%) and albumen (>90%) generates distinct T2-weighted signal intensities: hyperintense signals in the aqueous albumen versus hypointense signals in the lipid-rich yolk. Transverse relaxation time disparities significantly enhance image contrast, enabling clear delineation of yolk-albumen interfaces and internal structures, which provides a technical foundation for precise yolk feature extraction.

Building upon these findings, this study aims to: (1) Develop a non-destructive yolk percentage assessment method using 3.0 T MRI, with an automated feature extraction process and a high-precision prediction model applicable to all eggshell colors; (2) Validate the model’s applicability across different breeds and preservation conditions, and analyze the genetic parameters of yolk percentage; (3) Evaluate the impact of MRI scanning on hatching performance, providing technical support for breeding high-yolk-percentage hens and advancing innovation in the egg processing and breeding industries.

## MATERIALS AND METHODS

### Ethical Statement

The animal experiments in this study followed the Guidelines for Experimental Animals provided by the Animal Care and Use Committee of China Agricultural University, with permit number AW30405202-1-02.

### Experiment 1: Feasibility MRI Verification and Field Strength Optimization for Yolk Percentage Detection

#### Multi-field Strength MRI Scanning Parameter Settings

To investigate the effects of different field strengths on egg imaging, we employed three MRI systems with field strengths of 1.5 T (MAGNETOM ESSENZA Galaxy, Siemens, Erlangen, Germany), 3 T (uMR 770, United Imaging, Shanghai, China), and 7 T (R16 MRI System, Varian, California, USA) for the scanning of egg samples. Detailed scanning parameters for each field strength are presented in supplementary Table 1. All scanning data were exported in DICOM (Digital Imaging and Communications in Medicine) format and processed using 3D Slicer (version 5.4.0 r31938, Brigham and Women’s Hospital, Harvard Medical School, USA; https://www.slicer.org).

The selection of optimal field strength required comprehensive evaluation of scanning time, single detection cost, and spatial resolution to establish a high-precision egg yolk percentage detection model. For Experiment 1, we scanned and subsequently broke out a total of 85 eggs (30 at 1.5 T, 54 at 3 T, and 1 at 7 T) for validation purposes.

### Extraction of region of interest

a. Image acquisition and preprocessing: After the scanned DICOM files were imported into 3D Slicer software, orthogonal views of the sagittal (Y-Z), coronal (X-Y), and axial (X-Z) planes were automatically generated. The gray scale contrast of the image was optimized and the background noise was effectively suppressed by adjusting the window width and position parameters in the Volumes module, providing a clear base for subsequent yolk structure segmentation. b. Egg yolk segmentation and 3D reconstruction: The yolk structure was segmented using the "Segment Editor" module. Given the distinct gray scale threshold ranges between albumen and yolk, a suitable threshold interval was determined by adjusting the pixel value parameter to preliminarily separate these components. Manual correction was then performed to ensure segmentation accuracy. Upon completion, the 3D visualization function was enabled to generate and visualize the yolk’s three-dimensional structure. c. Quantification of yolk volume and other parameters: After the yolk was finely segmented, the software automatically extracted and calculated key parameters, which included yolk volume, surface area, and maximum diameter (Feret diameter). This process enabled accurate quantification of yolk morphology and provided reliable data for non-destructive yolk percentage testing.

### Destructive testing to determine yolk weight and percentage

**Fig. 1:**
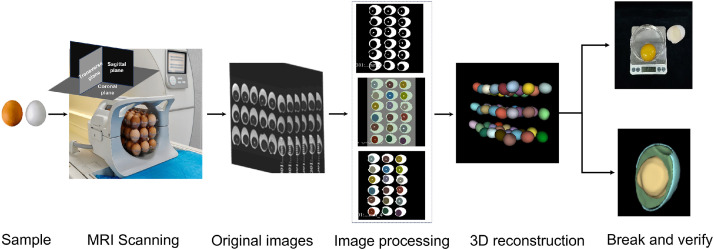
Schematic diagram of egg yolk image processing and analysis.

In order to verify the accuracy of the MRI non-destructive testing method, this study adopted the destructive testing method to establish the baseline data. The specific operation process was as follows: firstly, the weight of the whole egg was accurately determined using an electronic balance with an accuracy of 0.01 g; subsequently, the shell was broken and the yolk was separated using a yolk separator. Any residual albumen and chalaza on the yolk surface were then meticulously removed with absorbent paper. The yolk was weighed again immediately after this cleaning process. This weight served as the baseline reference for developing the prediction model based on non-destructive parameters, such as yolk volume; finally, yolk percentage was calculated by the formula "Yolk Percentage = (Yolk Weight / Whole Egg Weight) × 100%", and the results were compared with those obtained by MRI non-destructive testing to assess the reliability and accuracy of the non-destructive testing method. The complete test procedure is shown in Fig. 1.

### Experiment 2: Development and validation of a yolk weight prediction model

#### Collection of samples

Samples were collected from the core flock of Yukou Poultry, comprising eggs laid by 90 wks White Leghorn (WL) and Rhode Island Red (RIR) hens. A total of 360 WL eggs and 750 RIR eggs were collected over a 3-day period, resulting in a total sample size of 1110 eggs. All eggs were stored at 18-22°C and 40-70% relative humidity, and were uniformly subjected to MRI scanning and destructive testing on day 7 after collection. To ensure sample reliability, eggs with damaged shells or abnormal characteristics were excluded. Normal shape and size were defined according to Chinese National Standard SB/T 10638-2011 (Standardization Administration of China (SAC) 2011), which specifies criteria for egg appearance, size, and quality parameters.

### Sample processing

The study utilized a 3.0 T MRI device (uMR 770, United Imaging, Shanghai, China) to acquire egg images. A combined head/neck coil (Head & Neck Coil-24, United Imaging, Shanghai, China) was employed to optimize signal acquisition efficiency and enhance the imaging signal-to-noise ratio. The T2-weighted imaging technique was selected for scanning because the T2 sequence provides more significant signal contrast between yolk and the albumen. Specific scanning parameters are provided in table s1. All subsequent sample processing procedure followed the standardized procedure established in table s1.

### Modeling and validation

a. Data Cleaning. To ensure data reliability, the correlation between yolk weight and yolk volume was first examined. Since yolk density is relatively stable under normal physiological conditions, the ratio of yolk weight to yolk volume was calculated for each sample. The 3σ criterion was then applied to identify and exclude outliers with ratios exceeding ± 3 standard deviations from the mean. This step helped eliminate the influence of abnormal factors, such as measurement bias from chalaza residues and edge artifacts caused by magnetic field inhomogeneity. As a result, data accuracy and model validity were improved. After this process, 20 of 1110 samples (<2%) were identified as statistical outliers and excluded from further analysis.
b. Modeling and validation. After data cleaning, a yolk weight prediction model was developed and validated. A stratified random sampling strategy by breed was adopted to divide the samples into training and test sets at a 7:3 ratio. The training set was used for model development, while the test set was used for evaluation. The sample allocation is detailed in Table 1. Correlations between yolk weight and yolk volume, surface area, and maximum diameter were analyzed in the training set using Pearson correlation test. The significance of these correlations was assessed with t-tests. Based on linear regression analysis, the most strongly correlated parameter was selected to build the prediction model. The coefficient of determination (r²), root mean square error (RMSE) and mean relative error (MRE) were used to evaluate the model performance. All data were statistically analyzed using SPSS software (version 26.0, SPSS Inc., Chicago, IL), and plotted using OriginPro software (version 2024b, OriginLab Corporation, Northampton, MA).

**Table 1:** Division of egg samples.

Breed	Set	n	Egg Weight (g)	Yolk weight (g)	Yolk Volume (cm^3^)
WL	training set	243	58.97 ± 4.08	17.20 ± 1.36	16.70 ± 1.37
	test set	107	58.68 ± 4.36	16.95 ± 1.49	16.44 ± 1.42
RIR	training set	520	58.26 ± 4.38	14.94 ± 1.33	15.71 ± 1.36
	test set	220	58.44 ± 4.70	15.83 ± 1.41	15.09 ± 1.36

The table shows the division of egg samples. The samples of each breed were randomly divided into training and test sets according to 7:3, and results are expressed as mean ± standard deviation.

### Experiment 3: Application of the model

Following the development and validation of the yolk weight prediction model, this study systematically investigated the model's performance across diverse application scenarios, aiming to elucidate key determinants of yolk percentage variation and provide a theoretical basis for the breeding of laying hens.

### Analysis of differences in yolk percentage between breeds

To investigate the effect of breed factors on yolk percentage, the constructed model was used to compare yolk proportions between WL and RIR eggs in the test set. To eliminate potential interference from sample size imbalance, 107 samples were randomly selected from the RIR test set (original sample size n = 220) and compared between groups with the WL test set (n = 107). The significance of differences between groups was assessed using an independent samples t-test. Data normality was verified by the Kolmogorov-Smirnov test (*P* > 0.05). Difference histograms were plotted using OriginPro software (version 2024b, OriginLab Corporation, Northampton, MA) to visualize the characteristics of yolk percentage distribution between breeds.

### Comparative Analysis of Yolk Percentage Variations Across Egg Freshness Gradients

In addition to breed, egg freshness is an important factor affecting yolk percentage. This study investigated the variation pattern of yolk percentage under different storage times. Fifty-four fresh RIR eggs were selected and underwent MRI scanning both on the day of collection and after seven days of storage. Yolk percentage at different time points were calculated and compared using the established model. The significance of intra-group differences was analyzed by the paired samples t-test, and the normality of the data was confirmed by the Kolmogorov-Smirnov test (*P* > 0.05). Difference histograms were plotted using OriginPro software (version 2024b, OriginLab Corporation, Northampton, MA) to visualize the effect of freshness on yolk percentage.

### Estimation of genetic parameters for yolk percentage

Heritability represents the proportion of phenotypic variation attributable to genetic factors. Evaluating heritability is essential in breeding programs, as it improves selection efficiency and accuracy, enabling maximum genetic progress within limited resources and time. Evaluating the heritability of yolk percentage is crucial for establishing laying hens that produce eggs with high yolk percentage. In this study, eggs were collected from a core flock of 90 wks RIR laying hens at Yukou Poultry. One egg per hen was obtained, totaling 411 eggs. All eggs were stored for 7 days under standardized conditions (temperature of 18-22°C and relative humidity of 40-70%) before undergoing MRI scanning and destructive testing. Egg weight (EW) was determined using an electronic balance. Yolk weight (YW) was determined using a non-destructive yolk weight prediction model developed in this study, and the percentage of yolk (PY) was calculated accordingly. Eggshell strength (ESS) was measured with a Model-II eggshell strength tester (Robotmation, Tokyo, Japan). Albumen height (AH), yolk color (YC), and Haugh unit (HU) were determined using an automated egg quality analyzer (Robotmation EMT-5200, Tokyo, Japan). For eggshell thickness (EST), samples were collected from both poles and the equatorial region of each egg, measured with a digital display micrometer gauge (Mitutoyo, Kawasaki, Japan), and averaged to obtain the final value. The DMUAI module of the DMU (v6-R5-2-EM64T) software was used to calculate the variance and covariance components for the eight traits described above. A single-trait model was used for heritability estimation, and a two-trait animal model was used for genetic correlation and phenotypic correlation analyses. Our heritability estimates were based on a single egg measurement per hen. While this approach has limitations, previous studies have indicated that yolk percentage exhibits moderate repeatability (ranging from 0.636 to 0.672), which may lend some support to the reliability of single measurements for estimating genetic parameters (Zhang et al., 2024). And correlation heatmaps were plotted using ChiPlot (https://www.chiplot.online/) (accessed on 16 May 2025).

### Experiment 4: Effect of MRI scanning on hatchability of breeding eggs

Fertile eggs were obtained from a pure line of 90 wks WL hens raised at the Experimental Base of Poultry Genetic Resources and Breeding, College of Animal Science and Technology, China Agricultural University. Sixty eggs without fecal contamination, visible deformities, or cracks were randomly selected from the same laying day and divided equally into an experimental and a control group (30 eggs per group). No significant difference in egg weight was observed between the two groups. The experimental group underwent 3.0 T MRI scanning on the day of collection; while the control group received no scanning treatment. After the scanning, both groups of eggs were surface-sterilized with 0.1% Neosporin dilution (prepared by diluting commercially available Neosporin in sterile distilled water) to prevent microbial contamination during incubation. The entire surface of each egg was sprayed with the solution and allowed to air dry for 5 minutes before placement into an incubator. Eggs were then incubated under the conditions described by Li (Li et al., 2022). After 21 days, hatchability and healthy chick rate were recorded and compared between the two groups. Since both are categorical variables, chi-square tests were used to analyze differences in hatchability and healthy chick rate. Stacked bar charts were generated using OriginPro software (version 2024b, OriginLab Corporation, Northampton, MA) to visualize the results.

## RESULTS

### Experiment 1: Comparison of MRI detection performance at different field strengths

#### Performance Evaluation and Optimal Field Strength Selection for MRI in Egg Yolk Percentage Detection

A comparative analysis of the MRI detection effects at three field strengths of 1.5 T, 3 T and 7 T showed that all three field strengths can effectively detect egg yolks. However, detection accuracy at 1.5 T was significantly lower than that at 3 T and 7 T. A comprehensive evaluation of both the number of samples that can be detected at a single time and the detection accuracy is presented in Table 2. Combined with table s1, 3 T system provided the smallest voxel size, which contributed to higher resolution. Concurrently, it offered the highest detection efficiency, capable of scanning approximately 140 eggs per hour. These results demonstrate that the 3 T field strength delivered the best overall performance in terms of both detection accuracy and operational efficiency. Therefore, the 3 T MRI system was selected for subsequent development of the yolk detection model.

**Table 2:** Comparison of MRI detection accuracy, scanning time, and cost for different field strengths.

Field Strengths (T^1^)	Accuracy Level^2^	Number^3^
1.5	Moderate	30
3	High	54
7	High	1

1 T: Tesla, the unit of magnetic field strength.

2 Accuracy Level: Defined based on spatial resolution as determined by voxel size. Smaller voxel sizes represent higher spatial resolution, enabling more precise delineation of yolk boundaries. Moderate accuracy (1.5 T scans) corresponds to a voxel size of approximately 1.4 × 1.4 × 0.6 mm, while high accuracy (3 T and 7 T scans) corresponds to voxel sizes <1.0 mm in each dimension.

3 Number: Maximum egg capacity per scan for each MRI system and the actual number of eggs used in Experiment 1.

### Sample Image Acquisition and Analysis Based on 3T Field Strengths

Based on the advantages of 3 T field strength in detection performance, this study further analyzed the results of sample MRI image acquisition. 3 T MRI instrument can complete non-destructive detection and three-dimensional modeling of yolks of 54 eggs in a single scanning. By taking advantage of the difference in T1 and T2 relaxation times caused by the difference in moisture and fat content between the yolk and the albumen, the yolk and the albumen showed a significant difference in signal intensity in the MRI images acquired at a field strength of 3 T. Using 3D Slicer software, the yolk region was accurately isolated through threshold segmentation and manual correction, generating a three-dimensional reconstruction of the yolk structure, as shown in Fig. 1. The software automatically calculated yolk volume, surface area, and Feret diameter. This technique clearly distinguishes internal egg structures and provides a reliable data foundation for accurate yolk percentage detection.

### Experiment 2: Development and validation of yolk weight prediction models

**Fig. 2:**
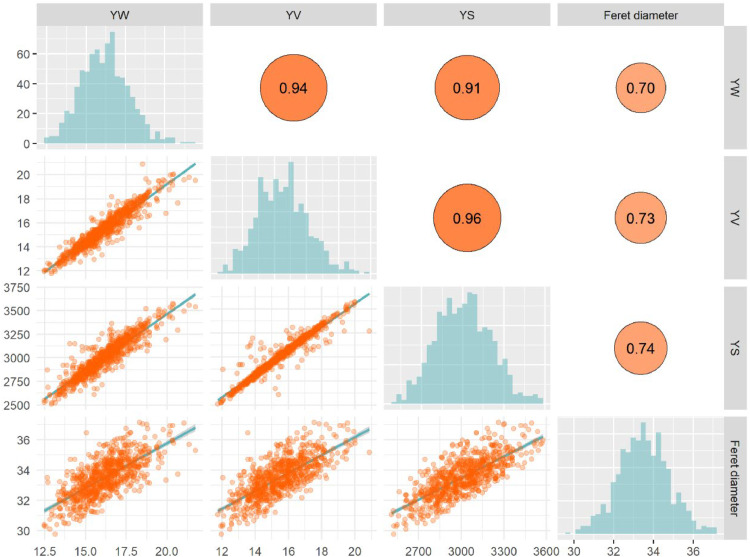
Scatterplot matrix between yolk volume, surface area, Feret diameter and yolk weight, with the lower triangles showing the scatter distributions and fitted curves between the variables, and the upper triangles labeled with Pearson's correlation coefficients.

As shown in Fig. 2, the pairwise relationship between yolk volume, surface area, Feret diameter and yolk weight was analyzed based on a matrix scatter plot. The lower triangular section displays scatter distributions and fitted curves for each variable pair, while the upper triangular section shows the corresponding Pearson’s correlation coefficients, quantifying the strength of each association. The results showed that yolk weight was significantly and positively correlated with yolk volume, surface area, and Feret diameter. That is, as yolk weight increased, its volume, surface area and Feret diameter increased synchronously. The fitted curves further confirmed linear relationships between these variables, supporting the use of linear modeling for parameter analysis. To explore the quantitative relationship between each parameter and yolk weight, three separate one-dimensional linear regression models were constructed for yolk weight with yolk volume, surface area, and Feret diameter as independent variables, respectively.

**Fig. 3:**
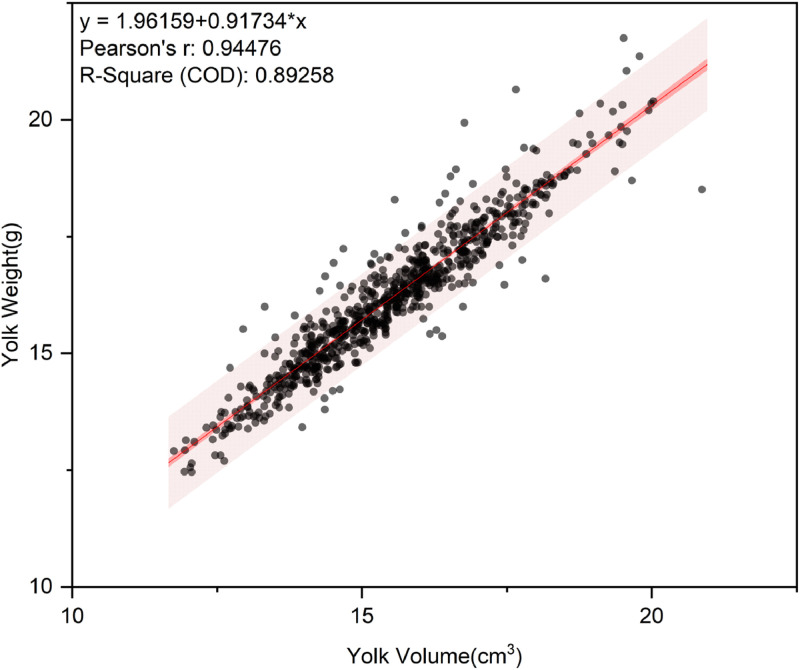
Prediction scatter plot of yolk weight by linear regression analysis model in training set. The solid red line is the linear regression line; the red shaded area is the 95 % confidence band, reflecting the stability of the regression line; the light red shaded area is the 95 % prediction band, demonstrating the range of fluctuations in the model's predicted values (*n* = 763).

The training set of 763 egg samples was analyzed using SPSS software to establish three separate univariate linear regression models for yolk weight with yolk volume, surface area and Feret diameter as independent variables, respectively. The results (Table 3) showed that all three independent variables were significantly positively correlated with yolk weight (*P* < 0.01). Among these, yolk volume demonstrated the strongest correlation with yolk weight (R = 0.945) and the model with yolk volume as the independent variable had the highest coefficient of determination (R² = 0.893), indicating that yolk volume had the best explanatory ability for yolk weight among the three NDT parameters. Based on these findings, a linear regression model for predicting yolk weight by yolk volume was developed as shown in Fig. 3.

**Table 3:** Linear regression models for predicting yolk weight from volume, surface area, and diameter in training eggs.

y	x	Model	R	r^2^
Yolk Weight	**Yolk Volume**	***Y*=1.962+0.917×_1_**	**0.945**⁎⁎	**0.893**
	Yolk Surface Area	*Y*=−4.758+0.007×_2_	0.914⁎⁎	0.836
	Feret Diameter	*Y*=−11.998+0.841×_3_	0.704⁎⁎	0.496

⁎⁎ *P* < 0.01.

The model constructed using the training set data required validation for its reliability and generalization ability using the test set. The yolk volume of the test set eggs was substituted into the established univariate linear regression model to predict yolk weight, and these predictions were compared with actual destructive measurements. As shown in Table 4, the model achieved a coefficient of determination (r²) of 0.907, indicating a good fit to the test set data. The root mean square error (RMSE) was 0.470, and the mean relative error (MRE) was 2.08%, demonstrating high prediction stability and minimal deviation. Compared with traditional detection methods, the model offers superior accuracy, meeting the demand for rapid and non-destructive detection of yolk weight in practical production and enabling accurate calculation of yolk percentage.

**Table 4:** Simple linear regression predictions for the test set.

Parameter	Model	r^2^	RMSE (g)	MRE (%)
Yolk Weight	*Y* = 1.962+0.917×_1_	0.907	0.470	2.08 %

**Fig. 4:**
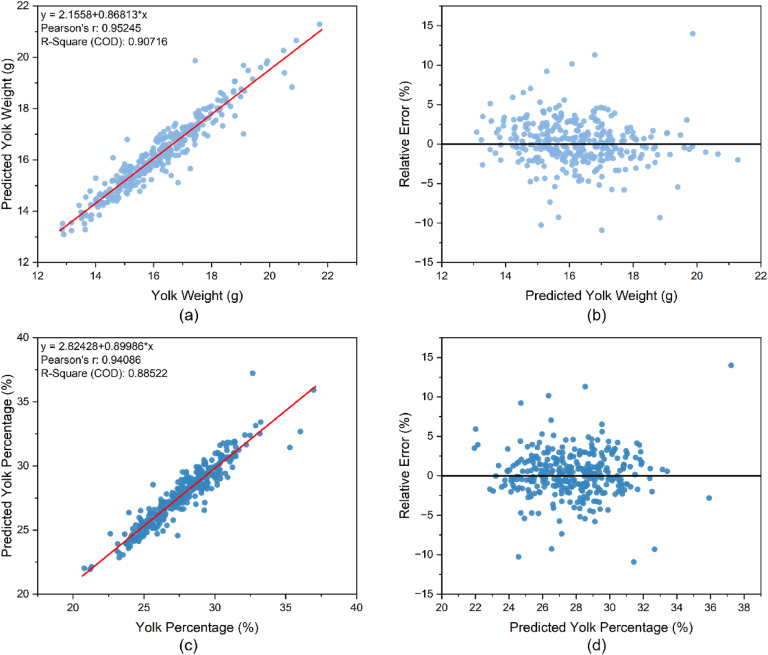
Predicted scatter plots and residual plots for the test set (*n* = 327), showing the relationship between predicted and actual yolk weight and yolk percentage, based on the simple linear regression model.

Figures 4(a) and (c) demonstrate the strong correlation between actual and predicted values of yolk weight and proportion, highlighting the model’s accuracy. In addition, the relative prediction error analysis, as shown in Fig. 4(b) and (d), showed that the relative errors of the majority of the observations were controlled within 10%, and the error points were randomly distributed on both sides of the zero error line without obvious patterns, indicating that the model predicted with high accuracy and the residuals conformed to the assumption of randomness.

### Experiment 3: Application of the model

#### Analysis of differences in yolk percentage between breeds

**Fig. 5:**
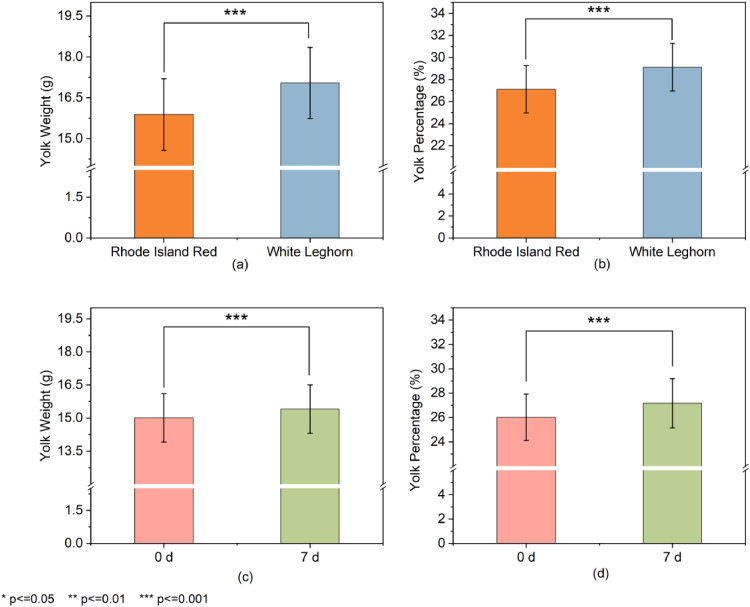
Comparative analysis of yolk weight and yolk percentage of eggs under different breeds and storage durations. (a) and (b): Yolk weight (g) and yolk percentage (%) of eggs from Rhode Island Red (RIR) and White Leghorn (WL) breeds (*n* = 107 per group); (c) and (d): Changes in yolk weight (g) and yolk percentage (%) of RIR eggs stored for 0 d and 7 d (*n* = 54 per group). Data are presented as mean ± SD. "***" indicates *P* < 0.001 from t-tests.

Based on the constructed yolk volume-yolk weight prediction model, a comparative analysis of yolk weight and yolk percentage between WL and RIR eggs was conducted to evaluate the model’s capability in distinguishing differences between breeds. As shown in Fig. 5(a) and (b), the yolk weight and yolk percentage of eggs from RIR hens were significantly lower than those of WL hens (15.88 ± 1.31 g vs. 17.05 ± 1.30 g, 27.12 ± 2.14% vs. 29.12 ± 2.16%, *P* < 0.001). The model successfully detected these significant breed-based differences, demonstrating its effectiveness as a non-destructive tool for comparative analysis. This capability confirms the model's applicability for testing eggs from different breeds and provides technical support for screening laying hen breeds with high yolk percentage.

### Analysis of differences in the proportion of egg yolks with different levels of freshness

To assess the model’s capability to detect changes associated with freshness, it was applied to compare yolk weight and percentage in RIR eggs at 0 and 7 days of storage. The results (Fig. 5(c) and (d)) showed a significant increase in both yolk weight and yolk percentage over the storage period (15.01 ± 1.10 g vs. 15.40 ± 1.10 g, 26.02 ± 1.91% vs. 27.17 ± 2.03%, *P* < 0.001). This successful detection of temporal changes demonstrates the model's sensitivity to freshness-related variations in yolk characteristics.

### Estimation of genetic parameters for yolk percentage

**Fig. 6:**
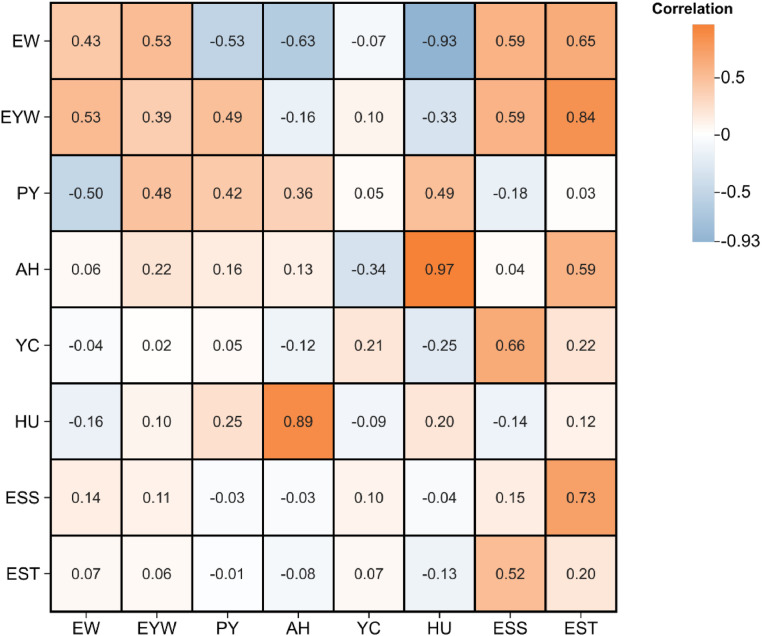
Genetic and phenotypic correlations between egg quality traits of Rhode Island Red. Abbreviations: EW, egg wight; EYW, egg yolk weight; PY, percentage of yolk; AH, albumen height; YC, yolk color; HU, haugh unit; ESS, eggshell strength; EST, eggshell thicknesses. The upper triangle represents genetic correlation, while the lower triangle represents phenotypic correlation, and the diagonal line shows the heritability of each trait. The color shades represent the heritability and the absolute size of the correlation, with orange indicating positive correlation and blue indicating negative correlation, which is convenient for intuitively judging the degree of correlation between traits.

Based on the yolk weight and yolk percentage data obtained by the NDT method in this study, the genetic parameters of egg quality traits in 90 wks RIR hens were estimated (Fig. 6). The results showed that the heritabilities of EW, YW and PY were 0.43, 0.39 and 0.42, respectively, indicating that PY is a trait with medium-high heritability under the conditions of this study, which suggests potential for improvement through genetic selection. In terms of phenotypic correlation, PY was significantly negatively correlated with EW (correlation coefficient-0.5), and positively correlated with YW, AH and HU (correlation coefficients 0.48, 0.16 and 0.25, respectively); in terms of heritability correlation, PY was strongly negatively genetically correlated with EW (correlation coefficient-0.53), and significantly positively genetically correlated with YW, AH and HU (correlation coefficients of 0.49, 0.36, 0.49, respectively). These results indicate that yolk percentage is closely related to several egg quality traits, and these genetic parameters can be used to develop a scientific selection strategy to accelerate the breeding process of egg breeds with high yolk proportions in the breeding practice of laying hens.

### Experiment 4: Effect of MRI scanning on hatchability of breeding eggs

**Fig. 7:**
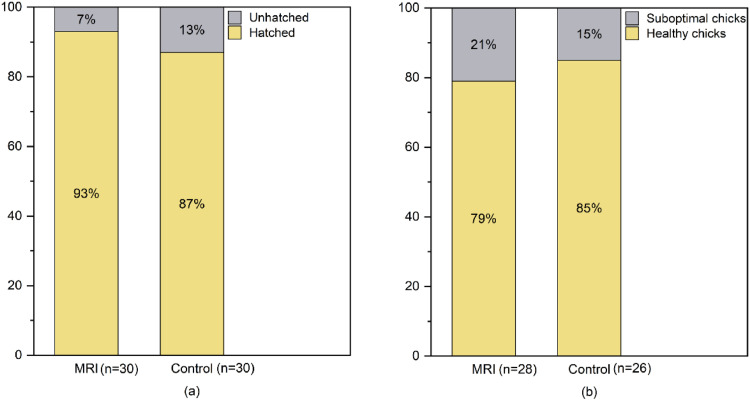
The effect of magnetic resonance scanning on chick hatchability and healthy chick rates.

To investigate the effect of MRI scanning on the hatchability of breeding eggs, the hatchability and healthy chick rates of breeding eggs in the MRI scanning and control groups were compared. As shown in Fig. 7, the hatching rate of the experimental group (MRI scanning group) was 93.3%, with a healthy chick rate of 88.7%, whereas the control group had a hatching rate of 90.0%, with a healthy chick rate of 86.7%. No significant differences were observed between the two groups in hatchability and healthy chick rate by chi-square test (χ² = 0.185 & 0.049, *P* > 0.05). Therefore, screening breeding eggs for appropriate yolk percentage using MRI scanning did not adversely affect hatching performance, demonstrating the feasibility of this protocol.

## DISCUSSION

As a core indicator of the nutritional density and economic value of eggs, accurate detection of yolk percentage is crucial for egg processing and hen breeding. The yolk weight prediction model developed using MRI technology in this study enables non-destructive and accurate detection of yolk percentage, providing an innovative solution for the industry.

Compared with existing non-destructive techniques, MRI offers distinct advantages due to its non-contact and fully automated 3D analysis capability. At the operational level, methods based on computer image analysis, require eggs to be placed in a darkroom and rotated multiple times to mitigate positional deviation, a process that can impede high-throughput screening (Kuchida et al., 1999); whereas MRI can simultaneously acquire 3D images of multiple eggs without complex sample preparation, significantly enhancing operational efficiency. In terms of model construction, previous studies have demonstrated the potential of multimodal fusion techniques for non-destructive prediction. For instance, they (Liu et al., 2024a) achieved promising results (R² = 0.82, RMSE = 1.05 g) for duck egg yolk weight prediction by combining spectral data, image information, and whole egg weight in a PLSR model. The approach presented in our study offers a distinct alternative based on a different principle. While models incorporating whole egg weight as a direct input parameter can be influenced by factors that alter total egg mass (such as storage conditions, breed, and hen age), our MRI-based method measures yolk volume directly by exploiting the inherent difference in water content between the yolk and the albumen. Since MRI signal intensity is influenced by proton density, it can distinguish these two compartments without relying on external metrics like whole egg weight. Consequently, the accuracy of our model is theoretically less susceptible to variability in overall egg weight, although this specific advantage should be rigorously validated in future studies with samples covering a wider range of factors. Furthermore, our model, constructed from a large dataset of 1110 eggs, achieved high goodness-of-fit (R² = 0.907, RMSE = 0.470 g, and MRE = 2.08 %), and automates the feature extraction process to minimize manual intervention and environmental interference. It is also noteworthy that while specialized techniques, such as the fuzzy logic algorithm for double-yolk egg recognition (Intarakumthornchai and Kesvarakul, 2020), effectively address the important task of egg type classification, our MRI-based approach provides a more comprehensive suite of information. It not only facilitates the identification of egg types based on yolk volume but also delivers precise quantitative data on internal compositional traits, such as yolk percentage. Together, the operational and modeling advantages discussed above were substantiated in our experimental setup. Collectively, these comparisons demonstrate the notable efficiency and accuracy advantages of MRI technology for the non-destructive assessment of egg yolk percentage in eggs from 90 wks hens.

The wide application of MRI technology in the field of food and agricultural products provides technical feasibility support for this study. Because MRI technology is non-radioactive and can visually describe the internal structural state and the distribution of protons in the measured individual, it has been widely applied in the quality assessment of food and agricultural products, such as vegetables and fruits, as well as in the non-destructive detection of internal damage (Zhou et al., 2021; Watanabe et al., 2023; Li et al., 2024). In recent years, the application of MRI in egg-related research has also been explored. For example, low-field nuclear magnetic resonance (LF-NMR) and MRI techniques were applied to non-destructively observe structural changes in internal moisture and other trends in salted quail eggs (Bao et al., 2020). This study further validated the effect of MRI on the hatchability of eggs. Within the scale of this validation (n = 30 per group), no significant difference in hatching rate was observed between the scanning group and the control group. When combined with both the MRI monitoring results of chicken embryo development (Chen et al., 2023) and its established industrial application for in-ovo sexing without compromising hatchability (Mcdougal, 2023), these findings collectively suggest that MRI technology shows strong potential to seamlessly link the egg screening and hatching processes, thus providing a technological safeguard for the breeding of high yolk percentage egg. Further studies with larger sample sizes would be beneficial to confirm these findings.

It has been shown that the yolk percentage is regulated by multi-dimensional factors such as the breed of laying hens, laying cycle, freshness and feed formulation (Ahn et al., 1997; Qiaoxian et al., 2020; Santos et al., 2022). In this study, based on the constructed non-destructive prediction model, we systematically analyzed the changing law of yolk percentage of eggs under different breeds and storage conditions. The results showed that the yolk weight and yolk percentage of eggs from RIR laying hens were lower than those from WL hens (15.88 g vs. 17.05 g & 27.12% vs. 29.12%, *P* < 0.001), this finding aligns with previous research (Zhang et al., 2024) on breed-specific yolk trait variations. Regarding the effect of freshness, the yolk weight of the eggs increased from 15.01 g to 15.40 g and the yolk percentage from 26.02% to 27.17% as the storage time was extended from 0 to 7 days (*P* < 0.001). This is attributed to a decrease in yolk membrane toughness and strength, and the transfer of water from the albumen to the yolk during storage, which is consistent with the previously reported results (Rouhollahi et al., 2024). The model in this study can accurately capture the above dynamic changes, which not only verifies the stability and accuracy of the model in different application scenarios, but also provides a reliable tool for monitoring the dynamics of egg quality.

Accurate evaluation of genetic parameters for egg quality is a key link between genetics theory and breeding practice, and is of dual significance for controlling raw material quality in the food processing industry and improving agricultural production efficiency. It has been shown that the heritability of yolk percentage, as an important egg quality trait, is usually in the middle level of 0.2 to 0.5 (Zhang et al., 2024), implying that this trait is significantly regulated by genetic factors. In this study, the yolk percentage of RIR laying hens at 90 weeks of age showed medium-high heritability (h^2^ = 0.42), which is consistent with the results of previous studies. However, it should be acknowledged that our estimates were based on measurements at a single time point (90 weeks), and these parameters may vary across different production stages. Further analysis of the genetic correlation between yolk percentage and other egg quality traits (e.g., EW, HU) suggested that breeding for higher yolk percentage could potentially improve the nutrient density of eggs, though the strength of these correlations may differ at peak production and post-peak periods compared to end of cycle. Future research should investigate these genetic parameters across multiple time points to provide a more comprehensive understanding of trait relationships throughout the production cycle.

This study systematically applied 3.0 T MRI technology to yolk percentage detection, constructed a non-destructive prediction model with high goodness of fit, and achieved a dual improvement in detection efficiency and accuracy. However, the industrialization and promotion of the research results are still facing practical challenges: the high acquisition and operation cost of 3.0 T MRI equipment (the cost of a single unit is over several million yuan, and the cost of a single inspection is about 23.15 yuan per piece) limits its popularization in large-scale production; Although the low-field MRI (<0.5 T) equipment commonly used in the food and agricultural industry has the advantages of portability and cost, it is difficult to meet the demand for measurement of fine structural parameters of egg yolk due to the lack of magnetic field strength, resulting in a lower signal-to-noise ratio and an image resolution of 1/3 of that of 3.0 T equipment. It is noteworthy that the prior research has explored artificial intelligence-based optimization of low-field magnetic resonance imaging, machine learning provides a new idea for breaking through the limitations of the equipment (Sorby-Adams et al., 2024), offering novel strategies to overcome current equipment limitations. Future advancements in algorithmic innovation and hardware improvement may enable high-precision application of low-field MRI in egg yolk percentage detection.

## CONCLUSION

This study developed and validated a non-destructive method for egg yolk percentage detection using 3.0 T MRI and medical imaging software, involving yolk segmentation, volume calculation, and regression modeling. The method demonstrated high accuracy, stability, and generalizability across eggs with varying shell colors and freshness levels. Furthermore, the yolk percentage data generated by this MRI-based method provided a non-invasive approach to estimate the heritability of this trait. The entire process was validated to be safe for breeding applications, with no adverse effect on egg hatchability. This approach establishes a novel framework for rapid, non-destructive egg quality assessment, providing critical technical support for genetic improvement of yolk percentage in laying hens.

## CRediT authorship contribution statement

**Bingxin Luo:** Formal analysis, Investigation, Methodology, Writing – original draft, Writing – review & editing. **Lin Xuan:** Conceptualization, Methodology, Writing – review & editing, Investigation. **Honglei Jin:** Methodology, Validation, Investigation. **Yuanhang Shi:** Investigation. **Jiahui Lai:** Investigation. **Runzhe Wang:** Investigation. **Longyu Zhuang:** Investigation. **Feiyu Chen:** Investigation. **Jiajie Yang:** Investigation. **Wenbin Zhou:** Investigation. **Anning Huang:** Investigation. **Guiyun Xu:** Funding acquisition, Resources. **Jiangxia Zheng:** Conceptualization, Funding acquisition, Resources, Supervision, Writing – review & editing.

## Disclosures

The authors declare that they have no known competing financial interests or personal relationships that could appear to influence the work reported in this paper.

## Appendix Supplementary materials

Image, application 1

## References

[bib0001] Ahmed M.W., Khaliduzzaman A.., Emmert J.L., Kamruzzaman M. (2025). An overview of recent advancements in hyperspectral imaging in the egg and hatchery industry. Comput. Electron. Agric..

[bib0002] Ahn D.U., Kim S..M., Shu H. (1997). Effect of egg size and strain and age of hens on the solids content of chicken eggs. Poult. Sci..

[bib0003] Bao Z., Kang D., Li C., Zhang F., Lin S. (2020). Effect of salting on the water migration, physicochemical and textural characteristics, and microstructure of quail eggs. LWT.

[bib0004] Chen L., Wang Z., Fu X., Wang S., Feng Y., Coudyzer W., Wu S., Zhang H., Chai Z., Li Y., Ni Y. (2023). Dynamic 3D morphology of chick embryos and allantois depicted nondestructively by 3.0T clinical magnetic resonance imaging. Poult. Sci..

[bib0005] Ciampa A., Dell’Abate M.T., Masetti O., Valentini M., Sequi P. (2010). Seasonal chemical–physical changes of PGI Pachino cherry tomatoes detected by magnetic resonance imaging (MRI). Food Chem..

[bib0006] Harnsoongnoen S., Jaroensuk N. (2021). The grades and freshness assessment of eggs based on density detection using machine vision and weighing sensor. Sci. Rep..

[bib0007] Hutchison M.J., Lirette A.., Etches R.J., Towner R.A., Janzen E.G. (1992). Research note: an assessment of egg yolk structure using magnetic resonance imaging. Poult. Sci..

[bib0008] Intarakumthornchai T., Kesvarakul R. (2020). Double yolk eggs detection using fuzzy logic. PLoS. One.

[bib0009] Klein S., Rokitta M., Baulain U., Thielebein J., Haase A., Ellendorff F. (2002). Localization of the fertilized germinal disc in the chicken egg before incubation. Poult. Sci..

[bib0010] Kuchida K., Fukaya M., Miyoshi S., Suzuki M., Tsuruta S. (1999). Nondestructive prediction method for yolk:albumen ratio in chicken eggs by computer image analysis. Poult. Sci..

[bib0011] Li L., Jia X., Fan K. (2024). Recent advance in nondestructive imaging technology for detecting quality of fruits and vegetables: a review. Crit Rev Food Sci Nutr.

[bib0012] Li J., Zhang X., Wang X., Sun C., Zheng J., Li J., Yi G., Yang N. (2022). The m6A methylation regulates gonadal sex differentiation in chicken embryo. J. Anim. Sci. Biotechnol..

[bib0013] Lindner T., Klose R., Streckenbach F., Stahnke T., Hadlich S., Kühn J.P., Guthoff R.F., Wree A., Neumann A.M., Frank M., Glass Ä., Langner S., Stachs O. (2017). Morphologic and biometric evaluation of chick embryo eyes in ovo using 7. Tesla MRI. Sci. Rep..

[bib0014] Liu Y.F., Xiao D..Q., Ni X., Li W.G. (2024). Estimating yolk weight of duck eggs using VIS-NIR spectroscopy and RGB images and whole egg weights. Poult. Sci..

[bib0015] Liu S., Yao Y., Wu N., Chen S., Xu L., Zhao Y., Tu Y. (2024). Development status and prospects of egg products industry in China. Food Sci. Anim. Prod..

[bib33] Mcdougal T. (2023, October). 5). Launch of MRI-based technology for in-ovo sexing of chickens. Poultry World.

[bib0016] Pathmanaban P., Gnanavel B.K., Anandan S.S. (2019). Recent application of imaging techniques for fruit quality assessment. Trends. Food Sci. Technol..

[bib0017] Qi L., Zhao M., Li Z., Shen D., Lu J. (2020). Non-destructive testing technology for raw eggs freshness: a review. SN. Appl. Sci..

[bib0018] Qiaoxian Y., Hui C., Yingjue X., Chenxuan H., Jianzhong X., Rongyan Z., Lijun X., Han W., Ye C. (2020). Effect of housing system and age on products and bone properties of Taihang chickens. Poult. Sci..

[bib0019] Réhault-Godbert S., Guyot N., Nys Y. (2019). The golden egg: nutritional value, bioactivities, and emerging benefits for Human health. Nutrients..

[bib34] Rouhollahi G., Mollazade K., Nourbakhsh H. (2024). Predicting the S-ovalbumin content and determining the freshness of chicken eggs via transmittance spectroscopy. Scientific reports.

[bib0020] Santos J.S., Araújo I.C.S., Martins P.C., Royer A.F.B., Café M.B., Andrade M.A., Uni Z., Stringhini J.H. (2022). The transfer of amino acids and minerals to the egg yolk and to the yolk sac of their progeny is affected by breeder age. J. Anim. Physiol. Anim. Nutr. (Berl).

[bib0021] Schechtman A.M., Knight P.F. (1955). Transfer of proteins from the yolk to the chick embryo. Nature.

[bib0022] Shi Y., Chen S., Liu Y., Liu J., Xuan L., Li G., Li J., Zheng J. (2024). Towards the perfect soft-boiled chicken eggs: defining cooking conditions, quality criteria, and safety assessments. Poult. Sci..

[bib0023] Soltani M., Omid M., Alimardani R. (2015). Egg quality prediction using dielectric and visual properties based on artificial neural network. Food Anal. Methods.

[bib0024] Sorby-Adams A.J., Guo J.., Laso P., Kirsch J.E., Zabinska J., Garcia Guarniz A.L., Schaefer P.W., Payabvash S., de Havenon A., Rosen M.S., Sheth K.N., Gomez-Isla T., Iglesias J.E., Kimberly W.T. (2024). Portable, low-field magnetic resonance imaging for evaluation of Alzheimer’s disease. Nat. Commun..

[bib0025] Standardization Administration of China (SAC) (2011).

[bib0026] Watanabe T., Sekiyama Y., Kawamura T., Fukuda Y., Nagata M. (2023). Tissue structural analysis for internal browning sweet potatoes using magnetic resonance imaging and bio-electrochemical impedance spectroscopy. J. Food Eng..

[bib0027] Yang S., Yang F., Dou W., Chi Y., Chi Y. (2024). Testing adulterated liquid-egg: developing rapid detection techniques based on colorimetry, electrochemistry, and interfacial fingerprinting. Food Chem..

[bib0028] Yuan L., Fu X., Yang X., Chen X., Huang G., Chen X., Shi W., Li L. (2023). Non-destructive measurement of Egg’s Haugh unit by Vis-NIR with iPLS-lasso selection. Foods..

[bib0029] Zhang J., Gao X., Zheng W., Wang P., Duan Z., Xu G. (2023). Dynamic changes in egg quality, heritability and correlation of these traits and yolk nutrient throughout the entire laying cycle. Foods..

[bib0030] Zhang X., Li Y., Li Q., Zhang T., Sun Y., Shi F., Chen J. (2024). Research note: genetic parameters estimation of egg quality traits in Rhode Island Red and White Leghorn chickens. Poult. Sci..

[bib0031] Zhou Y., Maître R., Hupel M., Trotoux G., Penguilly D., Mariette F., Bousset L., Chèvre A.M., Parisey N. (2021). An automatic non-invasive classification for plant phenotyping by MRI images: an application for quality control on cauliflower at primary meristem stage. Comput. Electron. Agric..

